# Two stage revision with a proximal femur replacement

**DOI:** 10.1186/s12891-019-2442-2

**Published:** 2019-02-08

**Authors:** Ralf Dieckmann, Tom Schmidt-Braekling, Georg Gosheger, Christoph Theil, Jendrik Hardes, Burkhard Moellenbeck

**Affiliations:** 1 Department of Orthopedics and Tumor Orthopedics, Muenster University Hospital, Albert-Schweitzer-Campus 1, 48149 Muenster, Germany; 2 Department of Tumor Orthopedics, Essen University Hospital, Hufelandstraße 55, 45147 Essen, Germany

**Keywords:** Periprosthetic infection, Proximal femur replacement, Two stage revision, Revision arthroplasty

## Abstract

**Background:**

Despite very good prosthesis retention times, the growing numbers of primary implantations of hip endoprostheses are leading to increasing numbers of revision operations. Periprosthetic infection, particularly in revision implants, often leads to a massive loss of bone stock, so that in a two-stage exchange the only option left is implantation of a megaendoprosthesis. This retrospective study investigated the clinical and functional outcome for patients who received megaendoprostheses in the proximal femur in two-stage exchange procedures.

**Methods:**

Forty-nine patients were treated between 1996 and 2014 (mean age 71 years, mean follow-up period 52 months). Microorganisms were isolated intraoperatively in 44 patients (89.9%). The reinfection rate was documented in patients who did not undergo any further revision surgery due to mechanical failure (primary) and in patients who had subsequent revisions after reimplantation and subsequent reinfection (secondary).

**Results:**

The mean C-reactive protein level at the time of reimplantation was 1.25 mg/dL (range 0.5–3.4). The primary success rate with curative treatment for prosthetic joint infection was 92% (four of 49 patients). The secondary success rate with infection revision cases was 82% (three of 17 revision cases). The mean Harris hip score was 69 (range 36–94). The majority of patients needed different types of walking aid or even wheelchairs, and only 50% of the patients were able to walk outside.

**Conclusions:**

Reinfections occurred in only 8% of patients who underwent two-stage exchanges with a proximal femur replacement. When revision surgery for the proximal femur replacement was required for mechanical reasons, however, the associated reinfections increased the reinfection rate to 18%. Proximal femur replacement achieves a clear reduction in pain, maintenance of leg length, and restoration of limited mobility, and the procedure thus represents a clear alternative to the extensive Girdlestone procedure, which is even more immobilising, or mutilating amputation.

## Background

The number of patients undergoing primary hip arthroplasty has increased over the last 10 years, reaching 160,484 cases in Germany in 2014 [1]. As many as 90% of the endoprostheses survive longer than 16 years, but 10% of the patients have to undergo revision surgery due to aseptic loosening, periprosthetic infections, or fractures. [1–3] Particularly for young patients who undergo primary arthroplasty, revision surgery is likely to become necessary later on. Each further operation required for the reasons mentioned, or attendant osteomyelitis in case of periprosthetic infection of the proximal femur, can contribute to bone loss. When there is extensive bone loss, with damage to or resection of the metaphysis, conventional revision stems are inadequate [4, 5]. Treatment options for reconstruction include the use of an allograft–prosthesis composite, resection arthroplasty, or proximal femur replacement [4–7]. All of the treatment options are associated with high complication rates and loss of function [5, 8]. Particularly in the treatment of periprosthetic infections, proximal femur replacement has the advantages that there are no allograft-to-host bone interfaces to heal, no problems of graft resorption, no fractures or disease transmission, and the reconstruction procedure is relatively easy [6, 7, 9, 10]. Nevertheless, the complication rate with proximal femur replacements in aseptic and septic revision cases is high [4]. The infection rate in proximal femur replacements carried out for non-oncological reasons is 8%, while with two-stage revisions the rate is 21.1% [4].

Previous studies have reported heterogeneous indications for the use of a proximal femur replacement. The present study only included patients with periprosthetic infection who needed a proximal femur resection. The aim of the study was to investigate the functional and clinical outcome after a two-stage procedure for reconstruction with a proximal femur replacement.

## Methods

### Patients and inclusion criteria

In a retrospective study, the functional and clinical outcome was analysed in 49 patients who underwent two-stage revision for prosthetic joint infection (PJI) and proximal femur defects. The inclusion criteria for the patients consisted of a minimum follow-up period of 6 months and a need for resection of the proximal femur due to a severe bone defect or osteomyelitis. The patients were treated in our orthopedic department between 1996 and 2014. There were 13 male and 36 female patients, with an average age of 71 years (range 37–85 years). The mean follow-up period was 52 months (range 6 months–13.5 years). The patients’ average body mass index was 28.1 (range 17.6–49.6).

### Clinical and functional follow-up

All of the patients received radiographic and clinical follow-up examinations. In case of death (*n* = 10), the last clinical and radiographic examination was evaluated. The indication for explantation and resection of the proximal femur and the numbers of previous operations and the reasons for them were recorded.

The functional outcome was evaluated during the outpatient examinations using the Harris hip score (HHS) [11]. In addition to the HHS, the patients were asked about the extent of their mobility in their everyday routines, with distinctions being made between whether the patients were bedridden, able to walk indoors or outdoors, and whether they needed crutches, a walking frame, or a wheelchair.

### End points and definitions

Up to 2012, PJI was diagnosed if at least one diagnostic method was positive in accordance with the Centers for Disease Control criteria [12]. Since 2012, we have used the Musculoskeletal Infection Society (MSIS) criteria [13]. The primary end point of this study was successful treatment of infection or reinfection involving loosening of the prosthesis. Clinical cure was defined as the patient having no clinical signs of inflammation, and negative C-reactive protein was assessed by the treating clinician at the date of the last available follow-up. Secondary end points were aseptic loosening of the stem, and death of the patients. Complications noted were aseptic loosening of the cup, dislocation, wound healing disturbances, and nerve damage.

### Surgical treatment

**Fig. 1:**
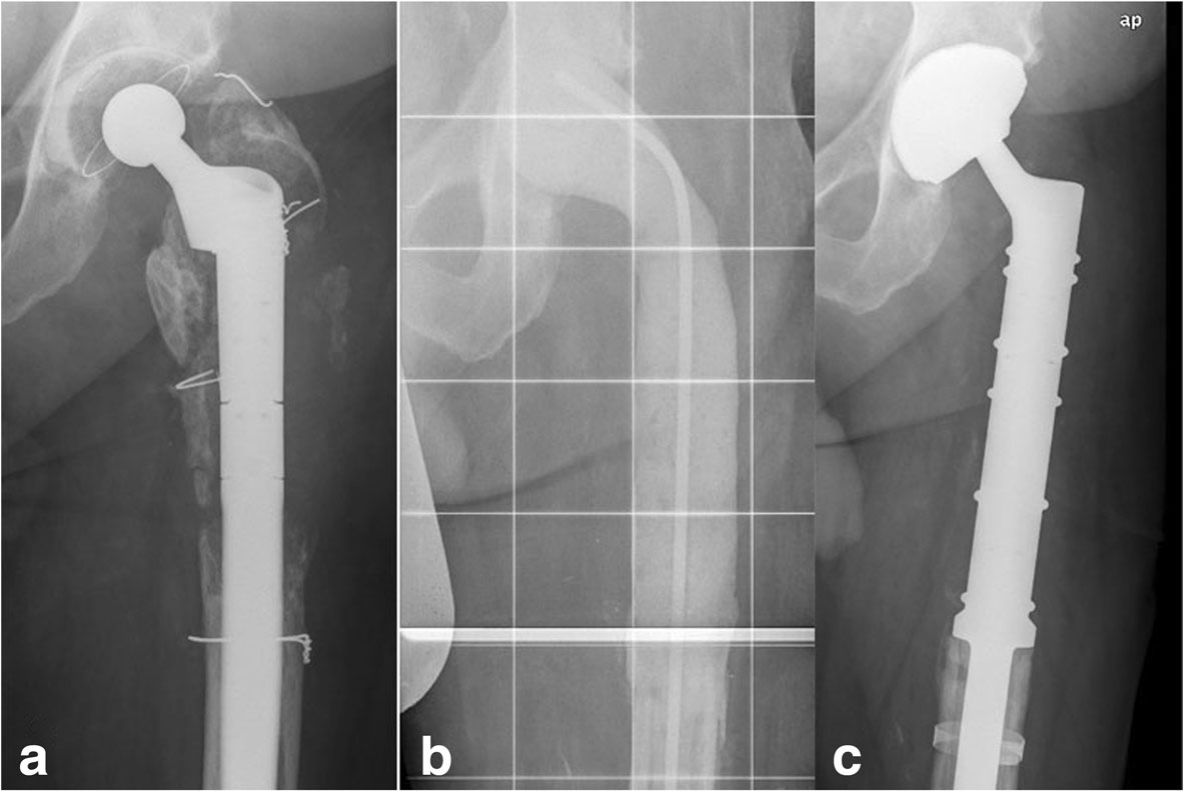
Defect in the proximal femur after arthroplasty. **a** Periprosthetic infection, with defect of the femur. **b** Spacer, with proximal femur resection. **c** Reimplantation of a proximal femur replacement and dual mobility cup (MUTARS system)

**Fig. 2:**
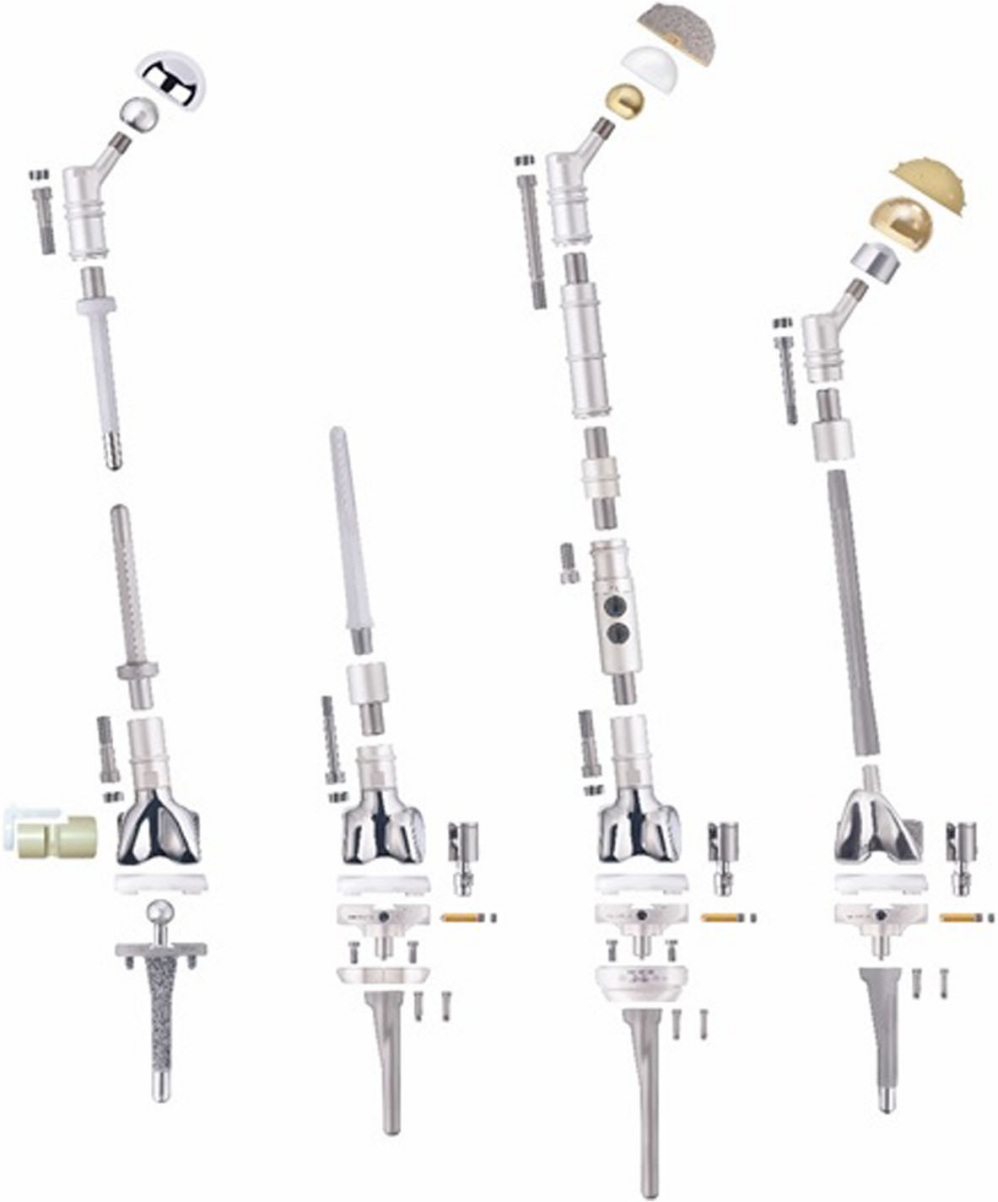
The MUTARS (Modular Universal Tumor and Revision System) for reconstruction of the femur

If at least one of the above-defined criteria [12, 13] was positive, a two-stage revision was performed. In the first step, the implant was removed, the necessary resection of the proximal femur was carried out, and an antibiotic-loaded polymethylmethacrylate (PMMA) spacer was implanted (Fig. 1). The composition of the antibiotics used in the spacer was adapted to the individual bacterial resistance (Table 1). Nearly all patients were treated for at least 2 weeks with parenteral antibiotic therapy, followed by oral antibiotic therapy for at least 4 weeks. An interval of 3 weeks between explantation and implantation of the new prosthesis only occurred in six cases. If the specific bacterium was not known, calculated antibiotic therapy with a third-generation cephalosporin and clindamycin was carried out. In other cases, specific antibiotic therapy was used. In case of persistent infection, another debridement and spacer exchange was done. The Modular Universal Tumor and Revision System (MUTARS; Implantcast GmbH, Buxtehude, Germany) for reconstruction of large bone defects was used for endoprosthesis reimplantation (Fig. 2). Cemented fixation was used with all of the MUTARS stems. After reimplantation, the patient received the original antibiotic therapy for 2 weeks parenterally, followed by 4 weeks of oral antibiotic therapy.

**Table 1:** Combinations used in the antibiotic-loaded polymethylmethacrylate (PMMA) spacers

Antibiotic combination	Patients (N)
Gentamicin	8
Gentamicin/vancomycin	5
Gentamicin/vancomycin/clindamycin	23
Gentamicin/clindamycin	8
Gentamicin/clindamycin/vancomycin/voriconazole	1
Gentamicin/clindamycin/vancomycin/meropenem	1
Gentamicin/clindamycin/daptomycin	1
None	2

After 2004, the MUTARS system became available with an antimicrobial silver coating, and this was used in most cases (*n* = 41). Due to the small numbers of patients with uncoated prostheses, meaningful statistical comparison with the silver-coated variant was not possible. The acetabular reconstruction was adapted to the defect (Table 2).

**Table 2:** Acetabular reconstruction

Acetabular reconstruction	Patients (N)
Dual mobility cup	22
Bipolar head	4
Constrained cup	5
Standard cup	4
Cage + dual mobility cup	13
Cage + constrained cup	1

### Statistical analysis

Statistical analysis was performed with IBM SPSS Statistics, version 24 (IBM Corporation, Armonk, New York, USA).

## Results

### Diagnosis of periprosthetic infections and microorganisms

The leading indications for explantation were fistula in 18 cases, bacteria identified on aspirated synovial fluid in 27 cases, intraoperative pus in three cases, and one patient with a positive leukocyte scan in combination with a large osteolysis.

Microorganisms were isolated intraoperatively in 44 patients (89.9%) (Table 3). *Staphylococcus epidermidis* was present in most cases.

**Table 3:** Microorganisms isolated intraoperatively

Microorganism	Patients (N)
*Acinetobacter baumannii* complex	1
*Candida albicans*	1
Corynebacteria	2
*Enterobacter cloacae*	2
*Enterococcus faecalis*	8 (1 VRE)
*Enterococcus faecium*	1
*Escherichia coli*	4
*Peptococcus* species	1
Propionibacteria	1
*Proteus mirabilis*	2
*Pseudomonas aeruginosa*	3
*Pseudomonas fluorescens*	1
*Salmonella* species	1
*Serratia marcescens*	1
*Staphylococcus aureus*	10 (5 MRSA)
*Staphylococcus capitis*	1
*Staphylococcus epidermidis*	18
*Staphylococcus haemolyticus*	2
*Staphylococcus hominis*	1
Sterile	5
*Streptococcus agalactiae*	1
Patients with two species	8
Patients with three species	3
Patients with four species	1

MRSA, methicillin-resistant *Staphylococcus aureus*; VRE, vancomycin-resistant enterococcus

The leading reasons for proximal femur resections were a failed osteosynthesis of the proximal femur in 16 cases, bone loss due to osteolysis or other reasons in 16 cases, 13 cases had a failed two-stage revision with osteomyelitis of the proximal femur and in 4 cases there was a periprosthetic fracture. On an average the patients have had 2,5 fold operations at the proximal femur before.

### Infection therapy

Spacer exchanges were necessary in 14 patients. The indications were persistent wound healing disturbance or persistent infection in six patients, and adaptation of the local antibiotic therapy in case of resistant bacteria in eight patients. One patient had infection spreading into the inguinal region. The mean C-reactive protein level at the time of reimplantation was 1.25 mg/dL (0.5–3.4 mg/dL).

The primary success rate with curative treatment for PJI was 92% (four of 49 patients). Reinfection occurred between 21 and 37 months after reimplantation (Table 4). The secondary success rate with infection in revision cases was 82% (three of 17 revision cases). Infection occurred in two patients in whom dislocations occurred and in one patient with a periprosthetic fracture.

Two-stage revisions were carried out in five patients with primary and secondary reinfections. One patient died of a metastatic malignancy before a two-stage procedure could be done, and one patient declined reimplantation.

### Complications

Dislocations were noted in six patients (12%). An open reduction of the hip was carried out in five of the patients and a closed reduction in one. Four of the six patients had dual mobility cups, one patient had a cementless revision cup, and one patient had a bipolar femoral head. The revision cup and the bipolar head were exchanged for cemented dual mobility cups. However, both patients had recurrent dislocations, and hip orthoses were adjusted. A Trevira tube was used in two patients. Extension of the prosthesis by about 1–2 cm was carried out in three patients. Secondary PJI occurred in two patients, and in one case a Trevira tube was used.

**Fig. 3:**
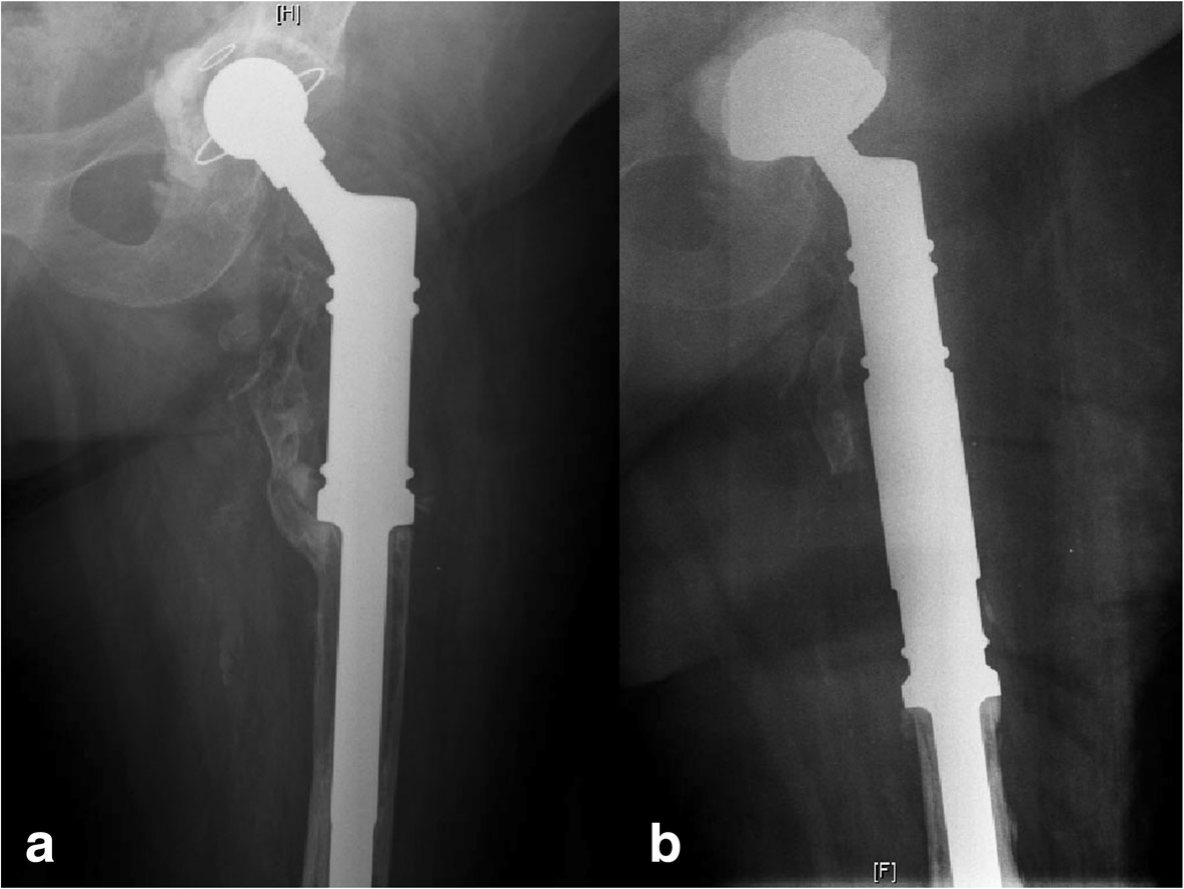
Aseptic loosening. **a** Preoperative state. **b** Postoperative state, with exchange of the cup and stem

**Fig. 4:**
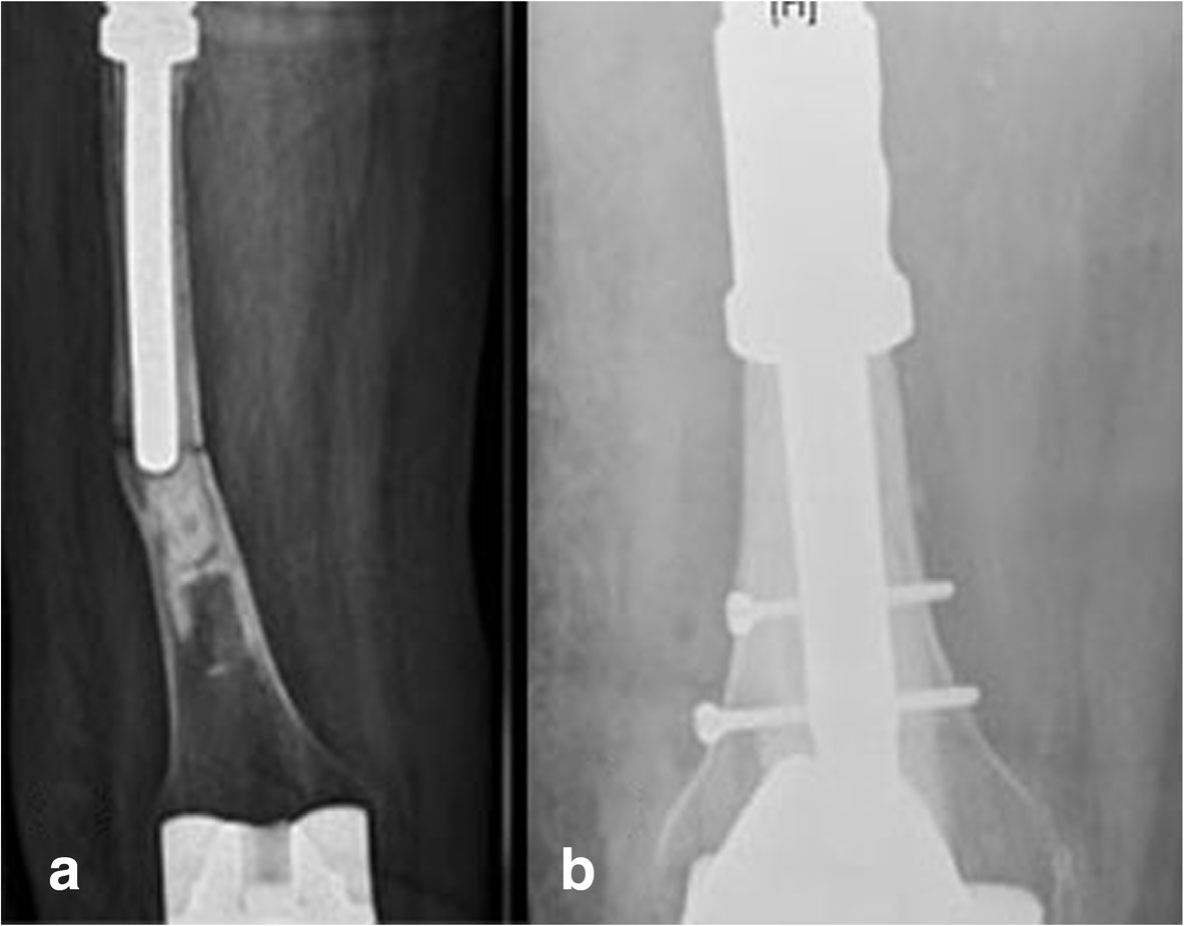
Periprosthetic fracture. **a** Preoperative state. **b** Postoperative state, with placement of a diaphyseal component

Aseptic loosening of the stem occurred in two patients, and an exchange of the stem was necessary (Fig. 3). A periprosthetic fracture occurred after a fall in one patient (Fig. 4). Further resection of the femur with elongation of the implant was necessary. A fatal PJI occurred after 1 year in this patient. Aseptic loosening of the cup occurred in four patients, making an exchange of the cup necessary. Wound healing disturbances occurred in six patients.

### Comorbidities and previous operations

Comorbidities were recorded for all of the patients (Table 5). The average number of previous operations was 2.49 (range 1–6). Eighteen patients (37%) had a history including femoral neck fractures or periprosthetic fractures.

**Table 4:** Patients with primary reinfections

Patient no., sex, age	History	Comorbidities	Bacterium	Spacer interval (days)	Complications	Time of reinfection (months)
1, F, 75	Arthroplasty in case of avascular necrosis of the femur head	–	*S. epidermidis*	34		21
2, F, 69	Periprosthetic hip fracture and stem revision	Diabetes mellitus, anticoagulation, obesity	*S. epidermidis*	78		29
3, F, 65	Osteosynthesis after neck fracture; secondary arthroplasty; stem exchange after loosening	Obesity, anticoagulation	*Propionibacterium avium*	84		23
4, F, 68	Cup exchange after loosening of primary arthroplasty	Chronic venous insufficiency	*S. epidermidis*	139		37

**Table 5:** Comorbidities

Comorbidity	Patients (N)
Anticoagulation	12
Obesity (body mass index > 30)	12
Renal insufficiency	6
Type 2 diabetes mellitus	4
Cancer	4
Cardiac insufficiency	3
Cirrhosis of the liver	1
Hepatitis B/C	2
Multiple sclerosis	1
Amyotrophic lateral sclerosis	1
Rheumatoid arthritis	1
Bacterial inflammation	6
Chronic venous insufficiency	
Patients with more than one comorbidity	13

### Functional results

Evaluation of the Harris hip score (HHS) was possible in 40 patients. One patient also had spastic diplegia; another patient had undergone amputation of the contralateral lower leg; five patients died before functional data could be recorded; and for two patients there were not enough data for the HHS. The mean HHS was 69 (range 40–94) (Table 6). However, all of the patients reached at least 40 points on the pain question.

**Table 6:** Harris hip score

Results	Patients (N)
Excellent (90–100)	3
Good (80–89)	6
Fair (70–79)	7
Poor (60–69)	19
Poor (< 60)	5

It was possible to record the patients’ everyday living routines in 44 cases, particularly in relation to whether patients were able to walk outdoors or only indoors, and what kind of aids they needed. Twenty-two patients were able to walk outdoors and 20 were able to manage everyday life at home. Only two needed permanent help. Three patients were able to walk without aids, 15 patients needed one crutch intermittently or constantly, 12 needed two crutches constantly, and 10 needed a walking frame. Two patients had to use wheelchairs — one due to spastic diplegia and the other because of a lower leg amputation on the contralateral side. Transfer from bed to wheelchair and for short distances with crutches was possible in both cases. Two patients were chair-bound and unable to reach their wheelchairs without help.

## Discussion

The age of patients receiving primary endoprostheses has been declining markedly in recent decades [2]. This has been due on the one hand to the good durability of the primary endoprostheses, as well as to increased expectations on the part of patients [2].

When the Swedish registry was analysed in 2009, the prosthesis survival rate after 16 years was 90% [14]. Despite these successes, however, 10% of the patients had to undergo revision procedures. The most frequent reasons for revision were aseptic loosening, followed by periprosthetic infection [14]. In the Norwegian prosthesis registry, the mean age of patients in whom stem revisions were carried out was 69 [15]. During the following 10 years, 25.6% of patients had to undergo surgery again due to aseptic loosening [15]. The continuing aging of the population, with average life expectancy now at over 80 years [16], means that another exchange operation can be expected in many patients.

In patients with aseptic loosening, a switch to a conventional revision stem is usually possible. However, following several stem revisions or osteomyelitic changes, massive segmental bone defects may develop in the proximal femur in which conventional anchoring of the stem is no longer possible. For reconstruction of segmental bone defects, either a megaendoprosthesis (Fig. 2) or an allograft composite prosthesis [6, 7, 17] is available. The use of a long stem to reconstruct elongated bone defects has been reported, but no long-term results for this method are available as yet [18].

The allograft composite prosthesis is mainly used in the Anglo-American countries in aseptic revision operations and in cancer treatment [19, 20]. In this reconstruction method, a cemented long stem prosthesis is introduced into a proximal femur allograft and the lower end is fixed into the residual femur using bone cement. It is postulated that the allograft will become integrated into the bone and that the musculature can be permanently reattached to the allograft, so that a better functional result can be achieved than with a megaendoprosthesis [21]. However, this method is only used in exceptional cases and with very small numbers of cases in two-stage revisions for periprosthetic infections [19, 20], since the nonvital foreign bone component is associated with a substantial risk of infection. Due to the high complication rates with allograft composite prostheses and allografts that are often difficult to obtain, the modular megaendoprosthesis has become the predominant method for reconstructing large bone defects in a two-stage procedure. The modular megaendoprosthesis has several advantages. The fact that both the length and also the antetorsion are freely adjustable after the introduction of the femoral stem means that the implantation technique is comparatively easy and standardised [17, 21–24]. The stem can be anchored with either a cement-free technique or with a cemented one [25]. The cement-free method is particularly advantageous in younger patients [25], but due to the age of the group of patients included in the present study, cement-free procedures were not possible in any of them. In older patients with multimorbid conditions, by contrast, use of the cemented technique can allow immediate full weight-bearing.

### Limitations

One possible limitation of the present study is its retrospective design, with the usual disadvantages in the collection of the data and observation of clinical courses. Another limitation is the sometimes short follow-up periods for the patients. However, complications such as luxation and periprosthetic infection usually emerge within a short period after the operation [5, 6, 26, 27]. It was possible to determine the functional outcome in particular at an early stage. One more possible limitation is that histopathological membrane classification was not routinely obtained [28]. This was due to the limited experience and not well-established relevance regarding the role of histopathology in the diagnosis of PJI at the time of the therapy of the patients.

### Infection treatment and control

When the indication was established, comparison between the two-stage exchange and the conventional modular revision stem in our department showed that no cases of low-grade infection were present [29]. Fistular situations were present in 37% of the cases. Major prior operations had consisted of osteosynthetic treatment for femoral neck fracture or periprosthetic fracture in 37% of the patients. Treatment of the infections was carried out in the same way as in the two-stage hip–total endoprosthesis (TEP) exchange, with resection of the proximal femur and implantation of an antibiotic-containing spacer. The composition of the antibiotics was adapted to the bacterial spectrum present in each case. It was found that repeated debridement and spacer exchanges were required to cure the infection in 29% of the patients.

In four of the 49 patients (8%), repeated primary infection occurred. In comparison with a two-stage hip–TEP exchange, in which infection control is reported to be up to 100%, this result can nevertheless be described as good in comparison with similar studies using a proximal femur replacement. However, it was also found that in cases in which revision was required, such as luxation, wound healing disturbances, cup loosening, and periprosthetic fracture, secondary infections developed in three of 17 patients (18%). In the literature, Shih et al. reported a reinfection rate of 50% in nine patients [30]. By contrast, Parvizi et al. reported an infection rate of 3% [5], and Sewell et al. a reinfection rate of 22% after two-stage exchanges [27]. A recent review by Korim et al. gives the mean infection rate as 21.1% [4]. Due to the comparatively high reinfection rate of 21.1% with proximal femur replacement reported in the literature, compared with 8% in our own group, and the low level of experience with one-stage exchanges in our department [23], the two-stage exchange with a temporary spacer appears to be preferable in patients with infection requiring proximal femur resection.

Due to the small number of cases included, a significant difference in the reinfection rate in patients with or without silver-coated prostheses was not observed. However, a trend towards a low infection rate was seen in comparison with the literature [4]. Hardes et al. have shown that there is a clear trend towards reduced infections in the field of proximal femur and tibia replacement when a silver-coated prosthesis is used (5.4%) in comparison with the traditional titanium prosthesis (19%) in patients with oncological conditions [31]. Whereas reinfections occurred in 38.5% of cases in the titanium group, all of the patients in the silver group were able to receive leg-preserving therapy [31]. However, longer-term results on this issue in the field of tumor oncology are still awaited.

### Complications unrelated to infection

The most frequent complication unrelated to infection was luxation, which occurred in 12% of the patients. The mean luxation rate reported in the literature is 18%, but it varies widely, from 0% to 42 [5, 8, 22–24, 26, 27, 30, 32–36]. In a meta-analysis, Korim et al. [4] showed that dislocation rates were significantly higher before 2000 [8, 35, 36] than after 2000 [5, 22, 24, 26, 27, 33, 34]. The authors do not offer any reason for this. Nor do they mention whether tripolar cups, dual head treatments, or large femoral head prostheses were used.

Hardes et al. reported that in aseptic revision cases with preservation of the standard cup without luxation protection, there was a luxation rate of 25% despite the use of an attachment tube [23]. On the one hand, these high luxation rates can certainly be explained by insufficient muscular control due to multiple previous operations [5, 8, 21, 35], as well as by the single cups that were often used in the past [4, 23]. One conclusion that was drawn from that study in our department was that all patients should receive a tripolar cup or a dual head. Since 2008, the attachment tube — due to its large plastic surface — has only been used in exceptional cases during reimplantation after a two-stage exchange.

Stem anchoring was cemented in all cases. Aseptic loosening occurred in two patients (4%). Malkani et al. [35] and Shih et al. [30] reported a loosening rate of 8%. Numerous other studies have reported loosening rates of 0%. [5, 22, 24, 26, 27, 32–34]. The two patients in the present study were treated with a repeat cemented stem exchange.

The rate of aseptic cup loosening, which occurred in four cases (8%), was comparable to that in similar studies [37–39]. The patients were treated successfully with a cup exchange using a Burch–Schneider antiprotusio-cage combined with a tripolar cup.

The periprosthetic fracture that occurred must be regarded as a misfortune and was independent of the prosthesis design. Stable stem anchoring was achieved by carrying out a stem exchange to a cemented diaphyseal component with screw locking to secure rotation.

### Functional results

The functional results, with a mean HHS of 69, are poorer in comparison with studies using a conventional two-stage exchange [29]. However, similar results are also obtained in comparable studies. Parvizi et al., for example, improved the preoperative HHS from a mean of 23.9 points to 64.9 [5]. Similar results were reported by Sewell et al., who increased the preoperative HHS from a mean of 28 points to 69 [27]. Nevertheless, in the present study — as in comparable groups — the majority of the patients were dependent on forearm crutches, walking frames, or for longer distances even wheelchairs [5, 8, 35]. However, mobility analysis showed that among 44 patients asked, 22 were able to carry out activities outside the home, despite an apparently low Harris hip score. The other half were able to look after themselves indoors.

## Conclusion

Using the two-stage exchange procedure with a MUTARS prosthesis makes it possible to achieve good cure rates of 92%. However, the rather poor functional results show that use of a proximal femur replacement in a two-stage exchange is associated with substantial functional limitations and must be regarded as an extremity-preserving procedure. Using the proximal femur replacement can achieve a marked reduction in pain, preservation of leg length, and restoration of limited mobility, and it is a clear alternative to the even more severely immobilising extended Girdlestone procedure [40] and mutilating amputation [41].

## Abbreviations

HHS: Harris-Hip-Score
MRSA: methicillin-resistant *Staphylococcus aureus*
MSIS: Musculoskeletal Infection Society
MUTARS: Modular Universal Tumor and Revision System
PJI: prosthetic joint infection
PMMA: polymethylmethacrylate
TEP: total endoprosthesis
VRE: Vancomycin resistant enterococcus
